# Depression among pregnant women and associated factors in Hawassa city, Ethiopia: an institution-based cross-sectional study

**DOI:** 10.1186/s12978-019-0685-x

**Published:** 2019-02-28

**Authors:** Bereket Duko, Getinet Ayano, Asres Bedaso

**Affiliations:** 1 Faculty of Health Sciences, College of Medicine and Health Sciences, Hawassa University, P. O. Box 1560, Hawassa, Ethiopia; 2 Department of reseaarch and training, Amanuel Mental Specialized Hospital, Addis Ababa, Ethiopia

**Keywords:** Depression, Antenatal care, Pregnant women, Ethiopia

## Abstract

**Background:**

Depression is the most prevalent psychiatric disorder during pregnancy. It is not only common and chronic among women throughout the world but also principal source of disability in pregnant women. The scarce information and limited attention to the problem might aggravate the consequence of the problem and can limit the intervention to be taken. The objective of the study was to assess the prevalence and factors associated with depression among pregnant women in public health institutions, Hawassa, Ethiopia.

**Methods:**

Institution based cross sectional study was conducted in May to July 2017. Pregnant women were selected by using systematic sampling technique. Data were collected through face-to-face interviews on socio-demographic, obstetric, psychosocial characteristics and depressive symptoms. Edinburgh Postnatal Depression Scale (EPDS) and Oslo Social Support Scale (OSS-3) were used to asses’ depressive symptoms and social support respectively. Descriptive and logistic regression analyses were carried out.

**Results:**

The mean age of the respondents was 23.82 ± (SD = 6.65) years. The prevalence of antenatal depression was 21.5%. When we adjusted for the effect of potential confounding variables, being in age group of 20–30 years [AOR = 5.85 (95% CI: (3.70, 10.14)], current pregnancy complication [AOR = 4.98 (95% CI: (3.01, 10.37)], unplanned pregnancy [AOR = 7.12, (95% CI: (3.12, 9.63)], categories of stressors (LTE) Health risk [AOR = 1.76, (95% CI: (1.01, 3.22)], previous history of depression [AOR = 2.76 (95% CI: (1.94, 6.75)], history of abortion [AOR = 1.52, (95% CI:1.04, 5.09)], history of still birth [AOR = 1.18, (95% CI: 1.08, 2.91)], poor social support [AOR = 2.14, (95% CI: 1.49, 3.11)] and poor baby father support [AOR = 3.21 (95% CI:1.93, 6.71)] were significantly associated with antenatal depression.

**Conclusion:**

For early detection and appropriate intervention, antenatal clinics should develop screening tools for depression during the routine antenatal care.

## Plain English summary

Among mental disorders that occur in pregnancy, depression has been now recognized as a global public health problem owing to its severity, chronic nature and recurrence as well as its negative impact on the general health of women and development of children. Thus, it is highly important to study the determinants of antenatal depression in pregnant mothers.

Pregnant mothers were asked their background characteristics, the people with whom they get social support, experience of stressful life events during the 6 months period prior to assessment and antenatal depressive symptoms.

A total of 317 pregnant women were interviewed. Pregnant mothers age ranged from 15 to 45 years, the majority were married, non-employed and earned less than 57 USD per month. 117 (36.9%) pregnant mothers had history of lost of new born child, 226 (71.3%) had good family feeling on current pregnancy and 214 (67.5%) had planned pregnancy.

In this study, 68 (21.5%) of pregnant mothers had antenatal depression. Mothers in the age group of 20–30 years, educational status, history of abortion, history of lose new born child, previous pregnancy complication, current pregnancy complication, partner support, family feeling and planed pregnancy were factors which are significantly associated with antenatal depression with *p*-value of < 0.05.

In summary, early detection and appropriate intervention is highly recommended. Antenatal clinics should develop screening tools for depression during the routine antenatal care.

## Background

Depression is a common mental disorder which is characterized by loss of interest, depressed mood, and disturbance of sleep, psychomotor activity, and difficulty to concentrate, guilty feeling, easily tiredness and recurring thought of death wish [1]. Among psychiatric problems that occur in pregnancy, depression is a prevalent mental health problem affecting about one in five women worldwide [2, 3].

It has been now recognized as a global public health problem owing to its severity, chronic nature and recurrence as well as its negative impact on the general health of women and development of children [4, 5]. The prevalence of antenatal depression is estimated to be 15.6% in low -and middle-income countries although there is no evidence from 92% of the countries [3].

Depression during the prenatal period in associated with a range of fetal and obstetric problem and adverse developmental child outcome [6], and in low and middle income countries evidence suggests that its impact extend beyond psycho-social developmental delay to child health outcome [7].

Studies conducted in low and middle income countries also showed that there was a significance association between antenatal depression and poor infant outcome like low birth weight, preterm delivery or both, fetal growth restriction [8]. Pregnant women with depression were also more likely suffer from obstetrical complication such as pre -eclampsia, uterine irritability& pregnancy induced hypertension [9].

Despite these known risk, depression during antenatal period was often not recognized and treatment rates were lower in pregnant than in non-pregnant women; even in well-resourced context [10]. It also affects the mother-infant interaction through its influence on the occurrence of post-natal depression [11].

Although depression has serious impact in pregnant mothers, per our literature review the research in this area is low. The aim of study was to determine the prevalence and factors associated with depression among pregnant mothers in Ethiopia.

## Methods

### Study setting and population

Institution based cross-sectional study was conducted from May to July 2017 at antenatal clinics in public health institutions in Hawassa, Ethiopia. Hawassa city, the capital of Southern Nations, Nationalities and Peoples Regional State (SNNPRS) which is located 273 KMs South of Addis Ababa, Ethiopia. The city is subdivided into 8 sub cities having a population of 343,175 (176,599 M, 166,576F) residents [12]. There are two governmental hospitals and five urban health centers. Among the mentioned health institutions, two health centers and one public hospital were selected randomly for the study. Pregnant mothers were allocated to their study institutions proportionally and were included in the study using systematic sampling technique (K = 2). Three pregnant mothers were excluded from the study.

### Inclusion and exclusion criteria

All pregnant women who had follow-up at their respective antenatal clinics were included in the study. Those who had severe form of mental illness that hinders their communication and critically ill women were excluded from the study.

### Data collection instruments

Demographic variables were collected using semi-structured questionnaire. Depressive symptoms were assessed using Edinburgh Postnatal Depression Scale (EPDS). It is a 10 item questionnaire, scored from 0 up to 3 (higher score indicating more depressive symptoms), that has been validated for detecting depression in ante partum and postpartum samples in many countries. The instrument was validated in Addis Ababa for postpartum use and showed sensitivity of 84.6% and specificity of 77.0% at the cutoff score 7/8 [13]. The cutoff point of EDPS among pregnant women is usually higher than postpartum women [14]. In this study, we used EPDS cutoff point of 13 to identify pregnant women with depressive symptom [15, 16]. Those pregnant women who scored > 13 were categorized as depressed women while pregnant women who scored below 13 were considered as non-depressed women [15]. Experience of stressful life events during the 6 months period prior to assessment was assessed using the List of Threatening Experiences (LTE) [17]. The 12 items were categorized into five categories namely health risks, loss of a loved one, relationship difficulties, income instability, and legal problems [18]. The LTE contains 12 categories of significant life events, for example relating to death of close persons, loss of relationships, imprisonment, and loss of valued object. These 12 categories accounted for two thirds of all events collected in the original development of the tool. It was reliable in the study (Cronbach’s α = 0.90) [19]. Social support was measured by the Oslo 3-item Social Support Scale. Oslo 3-item social support scale is 3-item questionnaire commonly used to assess social support. It has the sum score scale ranging from 3 to 14 with three broad categories: “poor support” 3–8, “moderate support” 9–11 and “strong support” 12–14. It was reliable in the study (Cronbach’s α = 0.88). The social support scores were categorized into poor or no social support for scores less than nine. The scores from 9 to 14 were considered moderate to strong support and merged together as “yes” for social support [20].

### Data processing and analyses

The coded data was checked, cleaned by entering into Epi Info version 7 and exported into Statistical Package for the Social Sciences (SPSS window version 22) for analysis. Descriptive statistics were employed to estimate the prevalence of antenatal depression. Binary logistic regression analysis was conducted to assess the relationship between each independent variable and dependent variable. The strength of association was measured by odds ratios with 95% confidence intervals. Statistical significance was declared at *P* < 0.05.

## Results

### Socio-economic and demographic characteristics

A total of 317 pregnant women were included. The mean age of the clients was 23.82 ± (SD = 6.65) years. The majority of participants were; 274 (86.4%) married, 155 (48.9%) Protestant, 202 (63.7%) non-employed, 110 (34.7%) of the respondents attended grade 11–12 and 216 (68.1%) earned less than 57 USD per month (Table 1).

**Table 1:** Frequency distribution of Socio-demographic and socio-economic factors among pregnant women at Public health institutions in Hawassa City, Ethiopia, 2017 (*n* = 317)

Variable	Category	Frequency (*n*)	Percent (%)
Age	15–19	108	34.1%
	20–30	171	53.9%
	> 30	38	12.0%
Marital status	Married	274	86.4%
	Divorced	34	10.7%
	Widowed	9	2.8
Religion	Protestant	155	48.9%
	Orthodox	99	31.2%
	Muslim	43	13.6%
	Catholic	20	6.3%
Education	No formal education	21	6.6%
	Grade 1–8	41	12.9%
	Grade 9–10	83	26.2%
	Grade 11–12	110	34.7%
	College/ university	62	19.6%
Occupational status	Government employee	34	10.7%
	Private employee	81	25.6%
	Housewives	202	63.7%
Monthly income	< 57 USD	216	68.1%
	57–96 USD	26	8.2%
	> 96 USD	75	23.7%

### Obstetric and clinical characteristics

Among the pregnant mothers, 110 (34.7%) of them had history of abortion, 117 (36.9%) had history of lost of new born child, 226 (71.3%) had good family feeling on current pregnancy, 104 (32.8%) had previous pregnancy complication, 214 (67.5%) had planned pregnancy, 173 (54.3%) had good partner support & 105 (33.1%) had current pregnancy complication (Table 2).

**Table 2:** Obstetrics and Social support factors among pregnant women at public health institutions at Hawassa City, Ethiopia, 2017 (*n* = 317)

Obstetric History and social support characteristics		Frequency	Percent %
History of abortion	Yes	98	34.7%
	No	219	65.3%
History of loss of new born	Yes	117	36.9%
	No	200	63.1%
Current pregnancy complication	Yes	105	33.1%
	No	212	66.9%
Pervious pregnancy complication	Yes	104	32.8%
	No	213	67.2%
Baby father support	Poor	141	54.6%
	Good	176	45.4%
Unplanned pregnancy	Yes	103	32.5%
	No	214	67.5%
Family feeling about current pregnancy	Good feeling	226	71.3%
	Poor feeling	91	28.7%
Categories of stressors (LTE) Health risk	Yes	120	37.9%
	No	197	62.1%
Social support	Good	176	55.5%
	Poor	141	44.5%
History of still birth	Yes	93	28.9%
	No	224	71.1%

### Prevalence of antenatal depression

The internal consistency of EPDS tool was acceptable (Cronbach’s α =0.87). The prevalence of antenatal depression (≥ 13 on EPDS score) was 68 (21.5%) (95% CI: 17.05, 25.10).

### Factors associated with antenatal depression

Finding from logistic regression analysis showed that age of the mother, educational status, history of abortion, history of lost of new born child, previous pregnancy complication, current pregnancy complication, partner support, family feeling, poor social support and planned pregnancy were significantly associated with depression with *p*-value of < 0.05. However, occupational status, history of chronic disease and history of low birth weight were not associated with depressive symptoms (Table 3).

**Table 3:** Factors associated with antenatal depression among pregnant women attending antenatal clinics in public health institutions in Hawassa, 2017 (*n* = 317)

Characteristics		Depression		COR at 95% CI	AOR at 95%CI
		Yes	No		
Age in years	Age group 15–19	11	97	1	**1**
	Age group 20–30	42	129	2.87 (1.89, 7.17)	5.85 (3.70, 10.14)*
	Age group > 30	16	22	6.41 (1.07, 9.95)	3.91 (0.83, 8.44)
Current pregnancy complication	Yes	55	50	15.58 (11.02, 21.55)	4.98 (3.01, 10.37)*
	No	14	198	1	1
Unplanned pregnancy	Yes	46	57	6.70 (4.51, 11.93)	7.12 (3.12, 9.63)*
	No	23	191	1	1
Categories of stressors (LTE) Health risk	Yes	12	108	2.32 (1.46, 5.48)	1.76 (1.01, 3.22)*
	No	9	188	1	**1**
Previous history of depression	Yes	13	35	4.89 (3.31,8.27)	2.76 (1.94, 6.75)**
	No	19	250	1	1
History of abortion	Yes	17	81	1.38 (1.01,4.51)	1.52 (1.04, 5.09)*
	No	29	190	1	1
History of still birth	Yes	15	78	1.34 (1.14, 3.41)	1.18 (1.08, 2.91)*
	No	28	196	1	1
Social support	Poor	24	94	3.37 (1.72, 6.85)	2.14 (1.49, 3.11)**
	Good	14	185	1	1
Baby father support	Good	17	159	1	1
	Poor	62	79	7.34 (3.52, 9.68)	3.21 (1.93, 6.71)*

1-Reference category, *- statistically significant –*P*-value < 0.05, **- statistically significant –*P*-value < 0.01

## Discussion

Based on Edinburgh Postnatal Depression Scale, the prevalence of depression among pregnant women attending antenatal clinics in the current study was 21.5% (95% CI: 17.05–25.10). This finding is in line with the study conducted in Nigeria [21], India [22], Ethiopia [9], Brazil [23], and rural Bangladesh [24]. On the other hand, the current finding is lower than the study conducted in South Africa [25], Jamaica [26] and London [27]. However, the finding is higher than the study conducted in Brazil [28], India [29], and Ethiopia [30]. The variation in prevalence might be due to methodological differences between studies & study setting (institution vs community based). The other difference might be due difference in data measurement tools. Our study used Edinburg postnatal depression scale while others used patient health questionnaire item nine (PHQ9), Hamilton depression scale (HDS) and beck depression inventory scale (BDI). The socio demographic and economic differences might also attribute for the difference in prevalence of antenatal depression between this study and the studies from other countries of the world.

Regarding age, being in age group of 20–30 years is 5.85 times [AOR = 5.85 (95% CI: (3.70, 10.14)] more likely to have antenatal depression than those in age group of 15–19 years. This is in line with other studies conducted in Nigeria [21] and in South Africa [25]. This might be supported by the fact that depression is commonly onset in the early 20’s of the life and most of the study participants were housewives who are financially dependent on their partner that could precipitate the occurrence of depression in this age group.

Having current pregnancy complication is 4.98 times [AOR = 4.98 (95% CI: (3.01, 10.37)] more likely to have depression. This finding is in line with other previous studies in low income countries [3, 30]. This might be due to the fact having complication by itself minimize their satisfaction to their life and this will impact their psychosocial wellbeing which is one of the possible causes of depression.

Like other similar studies, pregnant women who did not plan their current pregnancy 7.12 times [AOR = 7.12 (95% CI: (3.12, 9.63)] more likely to have depression when compared to those who planned their pregnancy [3, 23, 9, 15, 30]. It is established fact that pregnancy could cause physical, hormonal and psychological changes in pregnant mothers as a result it needs prior plan and preparation. Some studies in low-and-middle income countries showed that unplanned pregnancy is risk factor for antenatal depression.

The experience of stressful event related to injury and illness during the 6 months prior to the interview was 1.76 times [AOR = 1.76 (95% CI: (1.01, 3.22)] more likely to have antenatal depression. This finding was in agreement with other study findings [30, 31]. Having stressful situation prior to pregnancy or during pregnancy might predicted higher depression in the current pregnancy.

Having previous history of depression has significant association antenatal depression. Pregnant women who had past history of depression were 2.76 times [AOR = 2.76 (95% CI: (1.94, 6.75)] more likely to have antenatal depression as compared to those who had no history of past depression. This might be due to pregnant mothers are more biologically vulnerable to depression and in addition to this hormonal changes during pregnancy might precipitate previous depression or their psycho-social context may make them vulnerable to recurrent depression [3, 9, 24, 30, 32–34].

Previous history of abortion has significant association with antenatal depression. Those pregnant mothers who had previous history of abortion were 1.52 times [AOR = 1.53 (95% CI: (1.04, 5.09)] more likely to have depression. This finding was in-line with a study in Ethiopia [30] and Brazil [35]. This might be due to the thought of having abortion in the current pregnancy could result stressful situation that impose them to depression.

The study also revealed that, having history of still birth was 1.18 times [AOR = 1.18 (95% CI: (1.08, 2.91)] more likely to have antenatal depression. This is highly supported by other studies [3, 30, 36]. Pregnancies that occur after still birth often be caught with a change of emotions that osculates from fear and guilt to joy and relaxations. This might precipitate the obsession of residual sadness, not talking about it and withdraws the past events finally end up with isolation that might predispose to depression [37].

Regarding to social support, antenatal depression was significantly higher among pregnant mothers who had poor social support than women who had good social support. This might be due to having poor social support may lead to increased psychological distress and on the other hand, good social support is vital for those with good health in prevention of depression.

Lastly, women who had poor baby’s father support were found to be a statistically significant. This could be due to depressed women might assume that the support they receive is not sufficient enough or minimal. This finding is in line with other studies [3, 9, 16, 24, 25].

## Conclusion

In the current study, prevalence of depression among pregnant women attending antenatal clinics was high. Age group of 20–30, current pregnancy complication, unplanned pregnancy, categories of stressors (LTE) health risk, previous history of depression, history of abortion, history of still birth, poor social support and poor baby father support were significantly associated with antenatal depression. For early detection and appropriate intervention, antenatal clinics should develop screening tools for depression during the routine antenatal care.

### Strength and limitation of study

The study incorporated possible variables and used standardized measurement scales. However, being a cross-sectional study design; it did not allow establishing a temporal relationship between depression and other statistically significant variables. In addition, the study only included pregnant mothers who were started antenatal care follow up at health facilities this might over or under estimate the finding.

## Abbreviations

EPDS: Edinburg postnatal depression scale
LTE: Life threating events
OSS-3: Oslo Social Support Scale item 3
